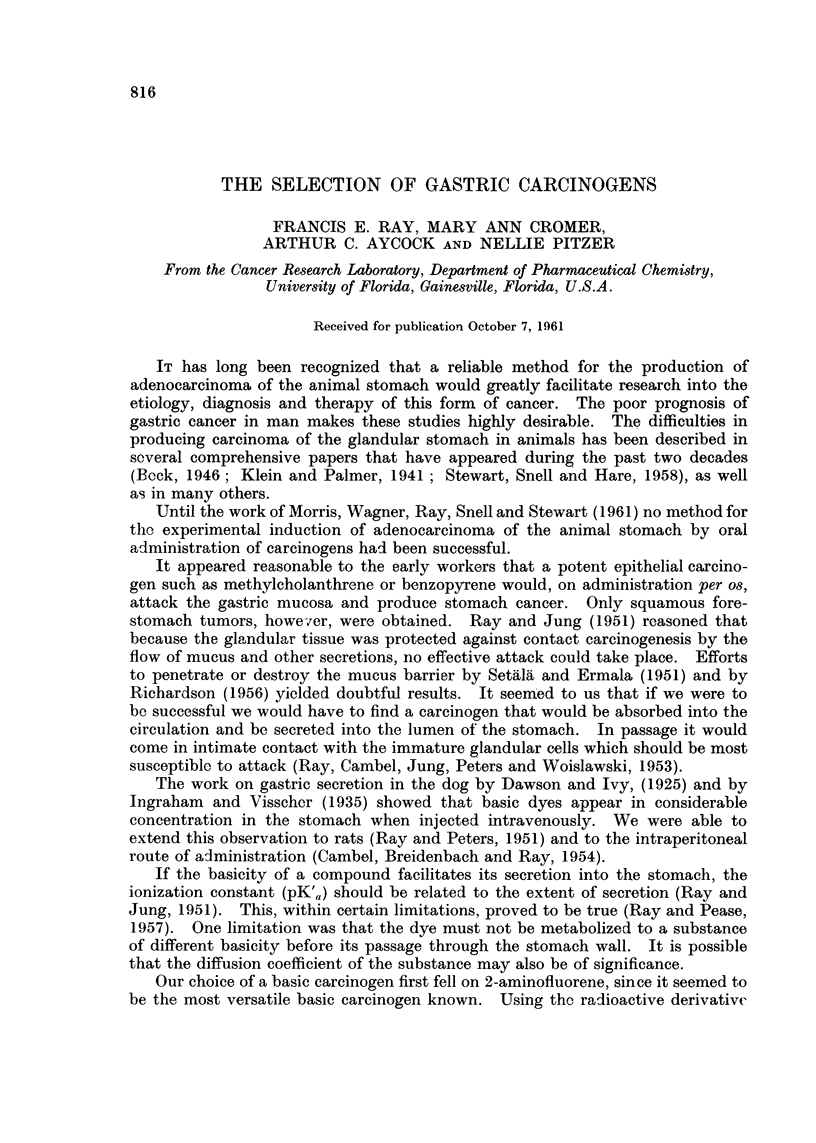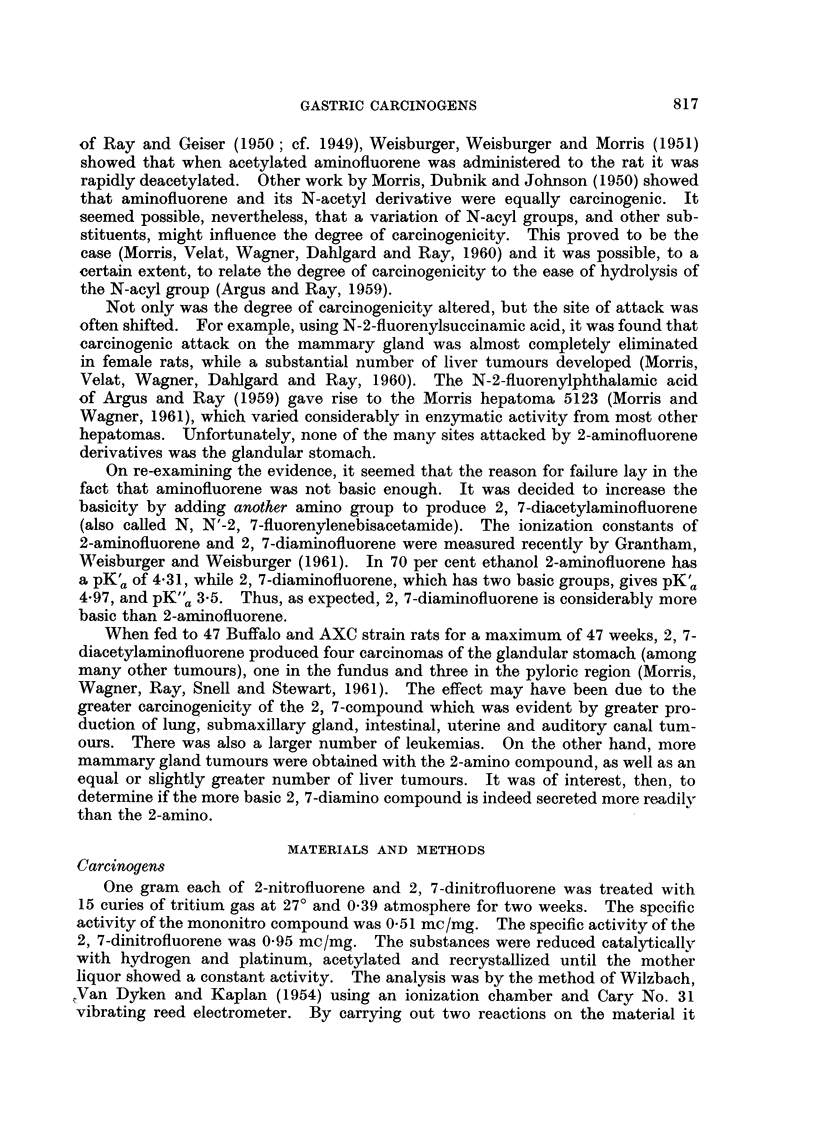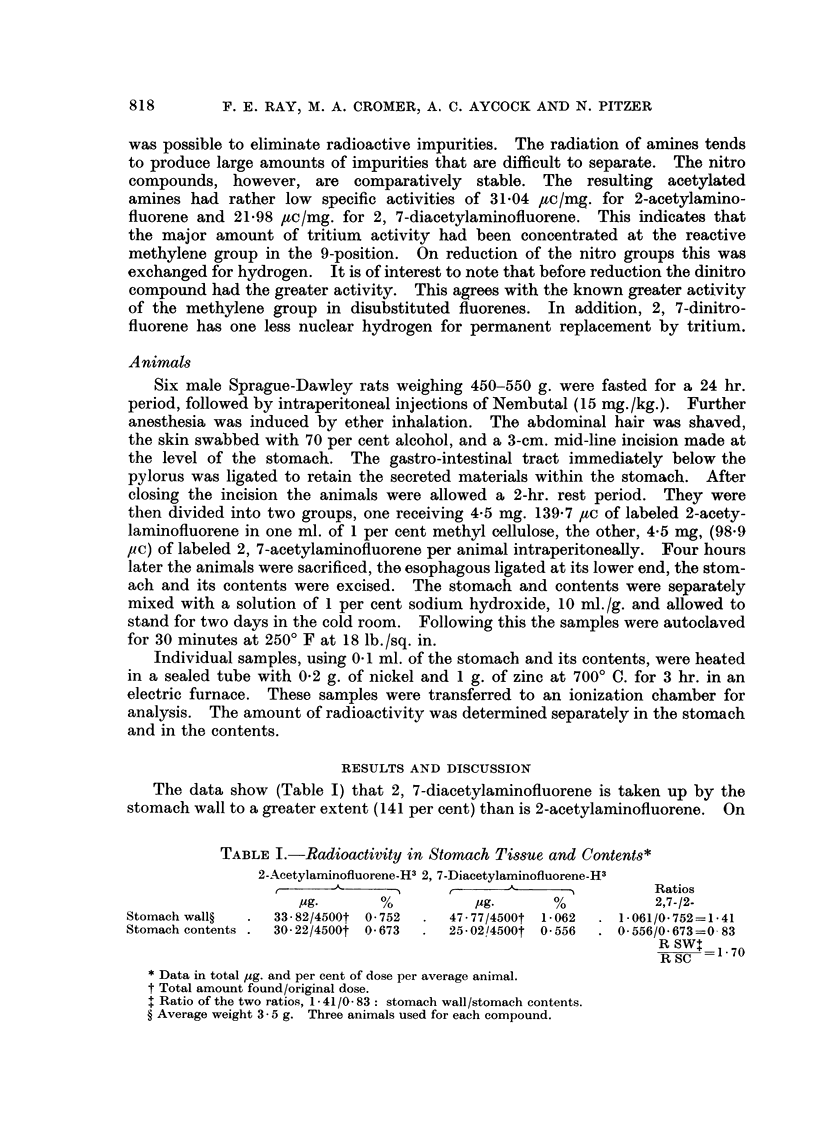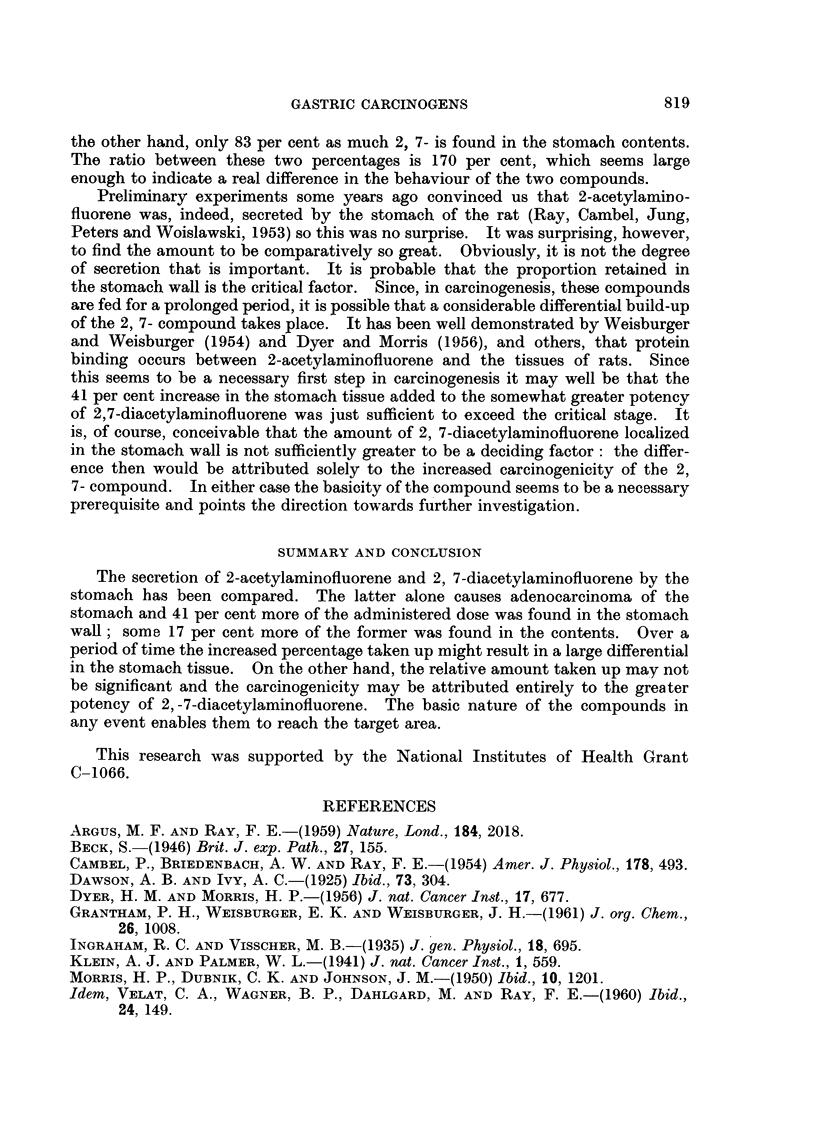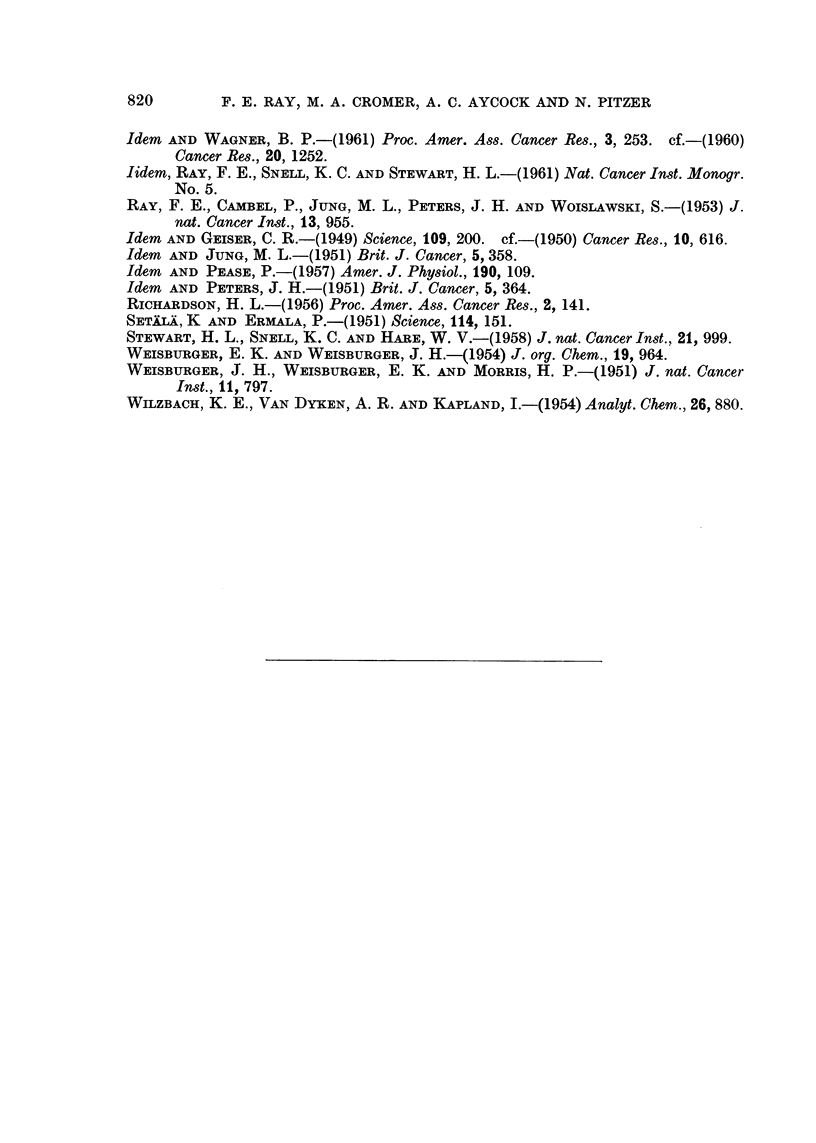# The Selection of Gastric Carcinogens

**DOI:** 10.1038/bjc.1961.93

**Published:** 1961-12

**Authors:** Francis E. Ray, Mary Ann Cromer, Arthur C. Aycock, Nellie Pitzer


### THE SELECTION OF GASTRIC CARCINOGENS

FRANCIS E. RAY, MARY ANN CROMER,
ARTHUR C. AYCOCK AND NELLIE PITZER

*From the Cancer Research Laboratory, Department of Pharmaceutical Chemistry, University of Florida, Gainesville, Florida, U.S.A.*

Received for publication October 7, 1961

It has long been recognized that a reliable method for the production of adenocarcinoma of the animal stomach would greatly facilitate research into the etiology, diagnosis and therapy of this form of cancer. The poor prognosis of gastric cancer in man makes these studies highly desirable. The difficulties in producing carcinoma of the glandular stomach in animals has been described in several comprehensive papers that have appeared during the past two decades (Bock, 1946; Klein and Palmer, 1941; Stewart, Snell and Hare, 1958), as well as in many others.

Until the work of Morris, Wagner, Ray, Snell and Stewart (1961) no method for the experimental induction of adenocarcinoma of the animal stomach by oral administration of carcinogens had been successful.

It appeared reasonable to the early workers that a potent epithelial carcinogen such as methylcholanthrene or benzopyrene would, on administration *per os*, attack the gastric mucosa and produce stomach cancer. Only squamous forestomach tumors, however, were obtained. Ray and Jung (1951) reasoned that because the glandular tissue was protected against contact carcinogenesis by the flow of mucus and other secretions, no effective attack could take place. Efforts to penetrate or destroy the mucus barrier by Setälä and Ermala (1951) and by Richardson (1956) yielded doubtful results. It seemed to us that if we were to be successful we would have to find a carcinogen that would be absorbed into the circulation and be secreted into the lumen of the stomach. In passage it would come in intimate contact with the immature glandular cells which should be most susceptible to attack (Ray, Cambel, Jung, Peters and Woislawski, 1953).

The work on gastric secretion in the dog by Dawson and Ivy, (1925) and by Ingraham and Visscher (1935) showed that basic dyes appear in considerable concentration in the stomach when injected intravenously. We were able to extend this observation to rats (Ray and Peters, 1951) and to the intraperitoneal route of administration (Cambel, Breidenbach and Ray, 1954).

If the basicity of a compound facilitates its secretion into the stomach, the ionization constant ($pK'_a$) should be related to the extent of secretion (Ray and Jung, 1951). This, within certain limitations, proved to be true (Ray and Pease, 1957). One limitation was that the dye must not be metabolized to a substance of different basicity before its passage through the stomach wall. It is possible that the diffusion coefficient of the substance may also be of significance.

Our choice of a basic carcinogen first fell on 2-aminofluorene, since it seemed to be the most versatile basic carcinogen known. Using the radioactive derivative of Ray and Geiser (1950 ; cf. 1949), Weisburger, Weisburger and Morris (1951) showed that when acetylated aminofluorene was administered to the rat it was rapidly deacetylated. Other work by Morris, Dubnik and Johnson (1950) showed that aminofluorene and its N-acetyl derivative were equally carcinogenic. It seemed possible, nevertheless, that a variation of N-acyl groups, and other substituents, might influence the degree of carcinogenicity. This proved to be the case (Morris, Velat, Wagner, Dahlgard and Ray, 1960) and it was possible, to a certain extent, to relate the degree of carcinogenicity to the ease of hydrolysis of the N-acyl group (Argus and Ray, 1959).

Not only was the degree of carcinogenicity altered, but the site of attack was often shifted. For example, using N-2-fluorenylsuccinamic acid, it was found that carcinogenic attack on the mammary gland was almost completely eliminated in female rats, while a substantial number of liver tumours developed (Morris, Velat, Wagner, Dahlgard and Ray, 1960). The N-2-fluorenylphthalamic acid of Argus and Ray (1959) gave rise to the Morris hepatoma 5123 (Morris and Wagner, 1961), which varied considerably in enzymatic activity from most other hepatomas. Unfortunately, none of the many sites attacked by 2-aminofluorene derivatives was the glandular stomach.

On re-examining the evidence, it seemed that the reason for failure lay in the fact that aminofluorene was not basic enough. It was decided to increase the basicity by adding *another* amino group to produce 2, 7-diacetylaminofluorene (also called N, N′-2, 7-fluorenylenebisacetamide). The ionization constants of 2-aminofluorene and 2, 7-diaminofluorene were measured recently by Grantham, Weisburger and Weisburger (1961). In 70 per cent ethanol 2-aminofluorene has a $pK'_a$ of 4·31, while 2, 7-diaminofluorene, which has two basic groups, gives $pK'_a$ 4·97, and $pK''_a$ 3·5. Thus, as expected, 2, 7-diaminofluorene is considerably more basic than 2-aminofluorene.

When fed to 47 Buffalo and AXC strain rats for a maximum of 47 weeks, 2, 7-diacetylaminofluorene produced four carcinomas of the glandular stomach (among many other tumours), one in the fundus and three in the pyloric region (Morris, Wagner, Ray, Snell and Stewart, 1961). The effect may have been due to the greater carcinogenicity of the 2, 7-compound which was evident by greater production of lung, submaxillary gland, intestinal, uterine and auditory canal tumours. There was also a larger number of leukemias. On the other hand, more mammary gland tumours were obtained with the 2-amino compound, as well as an equal or slightly greater number of liver tumours. It was of interest, then, to determine if the more basic 2, 7-diamino compound is indeed secreted more readily than the 2-amino.

### MATERIALS AND METHODS

### *Carcinogens*

One gram each of 2-nitrofluorene and 2, 7-dinitrofluorene was treated with 15 curies of tritium gas at 27° and 0·39 atmosphere for two weeks. The specific activity of the mononitro compound was 0·51 mc/mg. The specific activity of the 2, 7-dinitrofluorene was 0·95 mc/mg. The substances were reduced catalytically with hydrogen and platinum, acetylated and recrystallized until the mother liquor showed a constant activity. The analysis was by the method of Wilzbach, Van Dyken and Kaplan (1954) using an ionization chamber and Cary No. 31 vibrating reed electrometer. By carrying out two reactions on the material it was possible to eliminate radioactive impurities. The radiation of amines tends to produce large amounts of impurities that are difficult to separate. The nitro compounds, however, are comparatively stable. The resulting acetylated amines had rather low specific activities of 31·04 $\mu$c/mg. for 2-acetylaminofluorene and 21·98 $\mu$c/mg. for 2, 7-diacetylaminofluorene. This indicates that the major amount of tritium activity had been concentrated at the reactive methylene group in the 9-position. On reduction of the nitro groups this was exchanged for hydrogen. It is of interest to note that before reduction the dinitro compound had the greater activity. This agrees with the known greater activity of the methylene group in disubstituted fluorenes. In addition, 2, 7-dinitrofluorene has one less nuclear hydrogen for permanent replacement by tritium.

*Animals*

Six male Sprague-Dawley rats weighing 450–550 g. were fasted for a 24 hr. period, followed by intraperitoneal injections of Nembutal (15 mg./kg.). Further anesthesia was induced by ether inhalation. The abdominal hair was shaved, the skin swabbed with 70 per cent alcohol, and a 3-cm. mid-line incision made at the level of the stomach. The gastro-intestinal tract immediately below the pylorus was ligated to retain the secreted materials within the stomach. After closing the incision the animals were allowed a 2-hr. rest period. They were then divided into two groups, one receiving 4·5 mg. 139·7 $\mu$c of labeled 2-acetylaminofluorene in one ml. of 1 per cent methyl cellulose, the other, 4·5 mg, (98·9 $\mu$c) of labeled 2, 7-acetylaminofluorene per animal intraperitoneally. Four hours later the animals were sacrificed, the esophagous ligated at its lower end, the stomach and its contents were excised. The stomach and contents were separately mixed with a solution of 1 per cent sodium hydroxide, 10 ml./g. and allowed to stand for two days in the cold room. Following this the samples were autoclaved for 30 minutes at 250° F at 18 lb./sq. in.

Individual samples, using 0·1 ml. of the stomach and its contents, were heated in a sealed tube with 0·2 g. of nickel and 1 g. of zinc at 700° C. for 3 hr. in an electric furnace. These samples were transferred to an ionization chamber for analysis. The amount of radioactivity was determined separately in the stomach and in the contents.

### RESULTS AND DISCUSSION

The data show (Table I) that 2, 7-diacetylaminofluorene is taken up by the stomach wall to a greater extent (141 per cent) than is 2-acetylaminofluorene. On

TABLE I.—*Radioactivity in Stomach Tissue and Contents**

| | 2-Acetylaminofluorene-$H^3$ | | 2, 7-Diacetylaminofluorene-$H^3$ | | Ratios |
|---|---|---|---|---|---|
| | $\mu$g. | % | $\mu$g. | % | 2,7-/2- |
| Stomach wall§ | 33·82/4500† | 0·752 | 47·77/4500† | 1·062 | 1·061/0·752=1·41 |
| Stomach contents | 30·22/4500† | 0·673 | 25·02/4500† | 0·556 | 0·556/0·673=0 83 |
| | | | | | $\frac{\text{R SW}‡}{\text{R SC}}=1·70$ |

* Data in total $\mu$g. and per cent of dose per average animal.
† Total amount found/original dose.
‡ Ratio of the two ratios, 1·41/0·83 : stomach wall/stomach contents.
§ Average weight 3·5 g. Three animals used for each compound.

the other hand, only 83 per cent as much 2, 7- is found in the stomach contents. The ratio between these two percentages is 170 per cent, which seems large enough to indicate a real difference in the behaviour of the two compounds.

Preliminary experiments some years ago convinced us that 2-acetylaminofluorene was, indeed, secreted by the stomach of the rat (Ray, Cambel, Jung, Peters and Woislawski, 1953) so this was no surprise. It was surprising, however, to find the amount to be comparatively so great. Obviously, it is not the degree of secretion that is important. It is probable that the proportion retained in the stomach wall is the critical factor. Since, in carcinogenesis, these compounds are fed for a prolonged period, it is possible that a considerable differential build-up of the 2, 7- compound takes place. It has been well demonstrated by Weisburger and Weisburger (1954) and Dyer and Morris (1956), and others, that protein binding occurs between 2-acetylaminofluorene and the tissues of rats. Since this seems to be a necessary first step in carcinogenesis it may well be that the 41 per cent increase in the stomach tissue added to the somewhat greater potency of 2,7-diacetylaminofluorene was just sufficient to exceed the critical stage. It is, of course, conceivable that the amount of 2, 7-diacetylaminofluorene localized in the stomach wall is not sufficiently greater to be a deciding factor : the difference then would be attributed solely to the increased carcinogenicity of the 2, 7- compound. In either case the basicity of the compound seems to be a necessary prerequisite and points the direction towards further investigation.

### SUMMARY AND CONCLUSION

The secretion of 2-acetylaminofluorene and 2, 7-diacetylaminofluorene by the stomach has been compared. The latter alone causes adenocarcinoma of the stomach and 41 per cent more of the administered dose was found in the stomach wall ; some 17 per cent more of the former was found in the contents. Over a period of time the increased percentage taken up might result in a large differential in the stomach tissue. On the other hand, the relative amount taken up may not be significant and the carcinogenicity may be attributed entirely to the greater potency of 2, -7-diacetylaminofluorene. The basic nature of the compounds in any event enables them to reach the target area.

This research was supported by the National Institutes of Health Grant C–1066.

### REFERENCES

ARGUS, M. F. AND RAY, F. E.—(1959) *Nature, Lond.*, **184**, 2018.
BECK, S.—(1946) *Brit. J. exp. Path.*, **27**, 155.
CAMBEL, P., BRIEDENBACH, A. W. AND RAY, F. E.—(1954) *Amer. J. Physiol.*, **178**, 493.
DAWSON, A. B. AND IVY, A. C.—(1925) *Ibid.*, **73**, 304.
DYER, H. M. AND MORRIS, H. P.—(1956) *J. nat. Cancer Inst.*, **17**, 677.
GRANTHAM, P. H., WEISBURGER, E. K. AND WEISBURGER, J. H.—(1961) *J. org. Chem.*, **26**, 1008.
INGRAHAM, R. C. AND VISSCHER, M. B.—(1935) *J. gen. Physiol.*, **18**, 695.
KLEIN, A. J. AND PALMER, W. L.—(1941) *J. nat. Cancer Inst.*, **1**, 559.
MORRIS, H. P., DUBNIK, C. K. AND JOHNSON, J. M.—(1950) *Ibid.*, **10**, 1201.
*Idem*, VELAT, C. A., WAGNER, B. P., DAHLGARD, M. AND RAY, F. E.—(1960) *Ibid.*, **24**, 149.

*Idem* AND WAGNER, B. P.—(1961) *Proc. Amer. Ass. Cancer Res.*, **3**, 253. cf.—(1960) *Cancer Res.*, **20**, 1252.
*Iidem*, RAY, F. E., SNELL, K. C. AND STEWART, H. L.—(1961) *Nat. Cancer Inst. Monogr.* No. 5.
RAY, F. E., CAMBEL, P., JUNG, M. L., PETERS, J. H. AND WOISLAWSKI, S.—(1953) *J. nat. Cancer Inst.*, **13**, 955.
*Idem* AND GEISER, C. R.—(1949) *Science*, **109**, 200. cf.—(1950) *Cancer Res.*, **10**, 616.
*Idem* AND JUNG, M. L.—(1951) *Brit. J. Cancer*, **5**, 358.
*Idem* AND PEASE, P.—(1957) *Amer. J. Physiol.*, **190**, 109.
*Idem* AND PETERS, J. H.—(1951) *Brit. J. Cancer*, **5**, 364.
RICHARDSON, H. L.—(1956) *Proc. Amer. Ass. Cancer Res.*, **2**, 141.
SETÄLÄ, K AND ERMALA, P.—(1951) *Science*, **114**, 151.
STEWART, H. L., SNELL, K. C. AND HARE, W. V.—(1958) *J. nat. Cancer Inst.*, **21**, 999.
WEISBURGER, E. K. AND WEISBURGER, J. H.—(1954) *J. org. Chem.*, **19**, 964.
WEISBURGER, J. H., WEISBURGER, E. K. AND MORRIS, H. P.—(1951) *J. nat. Cancer Inst.*, **11**, 797.
WILZBACH, K. E., VAN DYKEN, A. R. AND KAPLAND, I.—(1954) *Analyt. Chem.*, **26**, 880.